# Arginine Enhances Osteoblastogenesis and Inhibits Adipogenesis through the Regulation of Wnt and NFATc Signaling in Human Mesenchymal Stem Cells

**DOI:** 10.3390/ijms150713010

**Published:** 2014-07-22

**Authors:** Jeong-Eun Huh, Jun-Young Choi, Ye-Ok Shin, Dong-Suk Park, Jung Won Kang, Dongwoo Nam, Do-Young Choi, Jae-Dong Lee

**Affiliations:** 1 East-West Bone & Joint Research Institute, Kyung Hee University, 149, Sangil-dong, Gangdong-gu, Seoul 134-727, Korea; E-Mail: jehuh2551@hanmail.net; 2 Department of Acupuncture and Moxibustion, College of Oriental Medicine, Kyung Hee University, 1, Hoegi-dong, Dongdaemun-gu, Seoul 130-701, Korea; E-Mails: 8416cjy@hanmail.net (J.-Y.C.); doctorkang@naver.com (J.W.K.); hanisanam@hanmail.net (D.N.); choi4532@unitel.co.kr (D.-Y.C.); 3 Department of Cancer Preventive Material Development, College of Oriental Medicine, Kyung Hee University, 1, Hoegi-dong, Dongdaemun-gu, Seoul 130-701, Korea; E-Mail: yogogiya@naver.com; 4 Department of Acupuncture and Moxibustion, Kyung Hee University Hospital at Kangdong, 149, Sangil-dong, Gangdong-gu, Seoul 134-727, Korea; E-Mail: dspark49@yahoo.co.kr

**Keywords:** arginine, osteoblastogenesis, adipogenesis, NFATc/Wnt signaling pathway, mesenchymal stem cells

## Abstract

Arginine, an α-amino acid, has been reported to exert beneficial effects that ameliorate health problems and prevent excessive fat deposition. In this study, we investigated whether the activation of cell signaling by arginine can induce osteogenic differentiation and modulate excessive adipogenic differentiation in human mesenchymal stem cells (MSCs). Arginine potently induced the expression of type Iα1 collagen, osteocalcin, and ALP in a dose-dependent manner without causing cytotoxicity. Arginine significantly increased the mRNA expression of the osteogenic transcription factors runt-related transcription factor 2 (Runx2), DIx5, and osterix. Furthermore, arginine demonstrated its antiadipogenicity by decreasing adipocyte formation and triglyceride (TG) content in MSCs and inhibiting the mRNA expression of the adipogenic transcription factors peroxisome proliferator-activated receptor γ (PPARγ), CCAAT/enhancer-binding protein α (C/EBPα), and fatty acid binding protein 4 (Fabp4). This effect was associated with increased expression of Wnt5a, and nuclear factor of activated T-cells (NFATc), and was abrogated by antagonists of Wnt and NFATc, which indicated a role of Wnt and NFATc signaling in the switch from adipogenesis to osteoblastogenesis induced by arginine. In conclusion, this is the first report of the dual action of arginine in promoting osteogenesis and inhibiting adipocyte formation through involving Wnt5a and NFATc signaling pathway.

## 1. Introduction

Human bone marrow mesenchymal stem cells (MSCs) are pleiotropic cells that differentiate into either adipocytes or osteoblasts [1,2]. Changes in the functional characteristics of the differentiation pathway of MSCs might contribute to the pathogenesis of osteoporosis [2]. Specifically, excessive adipogenesis is considered to be a major factor that negatively contributes to bone health and leads to bone loss [3]. Furthermore, a decrease in bone volume accompanied by an increase in adipose tissue has been observed in the bone marrow stroma of osteoporosis patients [4,5], which indicates that the differentiation pathways of osteoblasts and adipocytes are regulated jointly, and this implies that adipogenesis plays a critical role in bone loss [5,6].

Osteogenesis and adipogenesis, which share a reciprocal relationship in the bone marrow, are complex processes that include the proliferation of precursor cells and their commitment to a specific lineage and then terminal differentiation [6,7]. In MSCs, runt-related transcription factor 2 (Runx2), DIx5, and osterix are the main determinants of osteogenesis, whereas peroxisome proliferator-activated receptor-gamma-2 (PPARγ2), CCAAT/enhancer-binding proteins (C/EBPs) and fatty acid binding protein 4 (Fabp4) promote adipogenesis [8,9,10]. In osteoporosis, MSCs might be induced to differentiate into adipocytes rather than into osteoblasts [11,12]. Therefore, the enhancement of osteogenesis coupled with a concomitant reduction in adipogenesis could serve as a novel therapeutic target for treating osteoporosis. Although osteogenic and adipogenic transcription factors are recognized to play essential roles in determining the MSC differentiation associated with bone loss, the molecular mechanisms underlying the switch in the osteoblast/adipocyte differentiation of MSCs during aging remains to be understood.

Wnt signaling is a key pathway that controls bone formation and adipogenesis by regulating critical events such as cell-fate determination and cell proliferation and differentiation [13]. Wnt signaling is activated in osteoblast-committed MSCs, which further indicates that this pathway controls the balance between osteoblastogenesis and adipogenesis in the bone marrow [8,13]. Wnt signaling has been demonstrated to include both canonical (β-catenin-dependent) and noncanonical (β-catenin independent) pathways. The canonical Wnt/β-catenin pathway is activated through ligand-receptor binding, and then it induces osteoblastogenesis by upregulating Runx2 and inhibits adipogenesis by down-regulating PPARγ expression [9,14,15,16]. The noncanonical Wnt signaling pathway activated by Wnt5a involves several cascades of signal transduction, including the Wnt/c-Jun *N*-terminal kinase (JNK) pathway and the Wnt/calcium (Ca^2+^) pathway [13,17]. The activation of the Wnt/Ca^2+^ pathway ultimately leads to the binding of the nuclear factor of activated T-cells (NFATc) to specific DNA-binding sites [8,18,19]. These lines of evidence suggest that activation of Wnt signaling might prevent the increased adipogenesis that is associated with decreased osteogenesis in the aging bone.

Arginine is a major compound produced by *Astragalus membranaceus* Bunge, and it is one of the 20 most common natural amino acids [20]. In mammals, arginine is classified as a semi-essential or conditionally essential amino acid, depending on the developmental stage and the health status of the organism [20,21]. Oral administration of arginine for 2 weeks increases serum insulin-like growth factor I (IGF-I) levels and stimulates wound healing and immune functions in elderly people [22], and it also enhances the growth hormone (GH)-releasing activity of a synthetic hexapeptide (GHRP-6) in elderly and not young people [23]. Arginine can directly modulate the local production of IGF-I and enhance osteogenesis in mouse osteoblast-like MC3T3-E1 cells [24]. Arginine supplementation was recently reported to increase muscle gain and reduce the mass of body fat in growing-finishing pigs [25]. However, there are currently few reported for reducing adiposity in mammals, the detailed mechanisms of action of arginine remain to be elucidated.

In this study, we investigated whether arginine enhances osteogenic differentiation and inhibits adipocyte formation in MSCs by modulating osteogenic and adipogenic transcription factors and the Wnt signaling pathway.

## 2. Results and Discussion

### 2.1. Effect of Arginine on the Proliferation of MSCs

**Figure 1:**
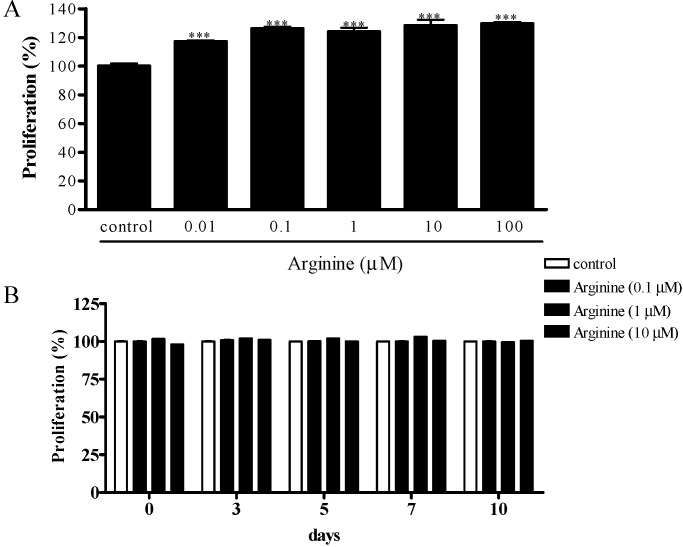
Effect of arginine on the proliferation of mesenchymal stem cells (MSCs). Cells were seeded in 96-well plates at a density of 2 × 10^4^ cells/well and allowed to attach for 12 hin growth medium. The cells were then treated with various doses of arginine (0.01–100 μM) for 48 h (**A**); or arginine (0.1–10 μM) for 3, 5, 7, and 10 days (**B**). Cell proliferation was assessed using Cell Counting Kit-8. Values are expressed as means ± S.E.M. of three independent experiments. *** *p* < 0.001 compared with control.

To examine how arginine affects cell proliferation, we treated MSCs with 0, 0.1, 1, and 10 µM arginine for 1, 3, 5, 7, and 10 days. Arginine dose-dependently enhanced cell proliferation after treatment for 48 h and increased the proliferation of cells in a statistically significant manner, by nearly 36%, at a concentration of 10 µM (Figure 1A). However, from days 3–10, arginine at doses ranging from 0.1–10 μM did not stimulate MSC proliferation, which suggests that arginine does not affect MSC proliferation at this stage (Figure 1B). These results extend the findings showing that arginine promotes both cell proliferation and differentiation and indicates that arginine acts on the lineage commitment of MSCs toward osteoblasts and adipocytes at a late stage.

### 2.2. Effect of Arginine on Osteogenic Differentiation of MSCs

**Figure 2:**
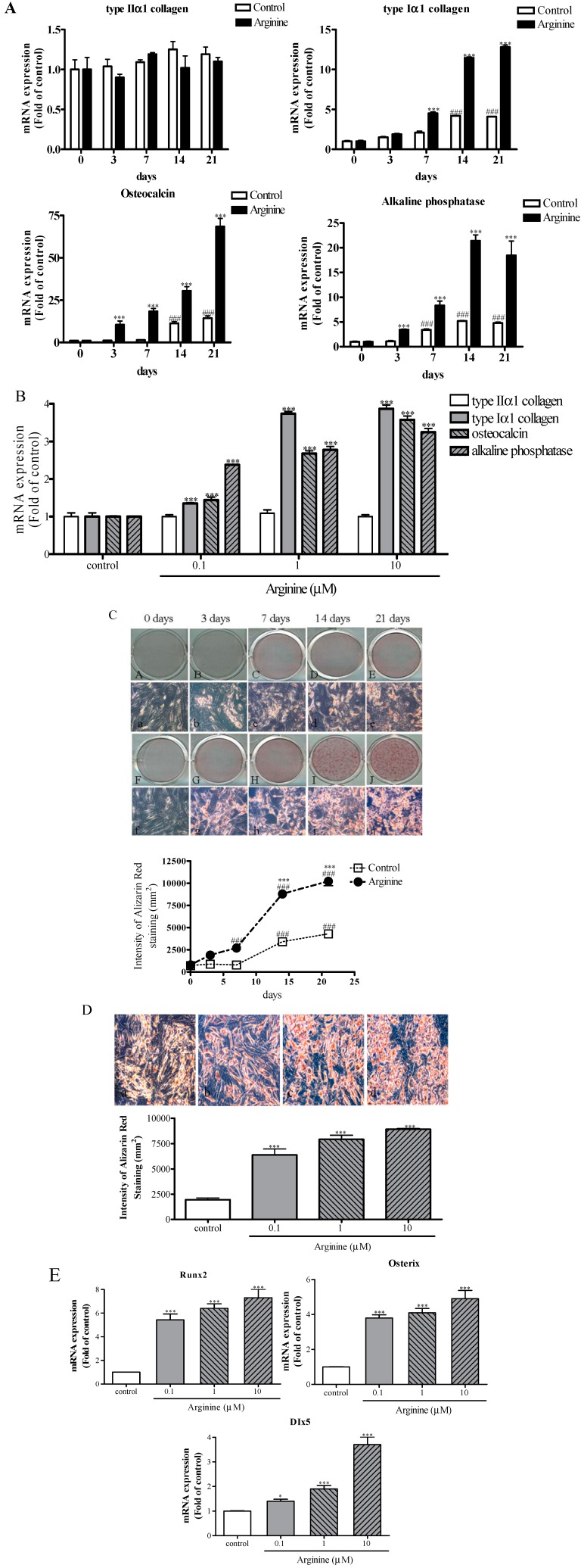
Effect of arginine on osteoblastogenesis in MSCs. (**A**) Time-dependent expression of osteogenic marker genes during the differentiation of MSCs. Osteogenic differentiation was induced in MSCs for 21 days by using arginine 1 μM under osteogenic conditions. The levels of mRNA expression of type IIα1 collagen, type Iα1 collagen, osteocalcin, and alkaline phosphatase were measured using qRT-PCR, and normalized relative to β-actin expression; (**B**) Dose-dependent effect of arginine on the mRNA expression of osteogenic markers; (**C**) Temporal response of arginine on mineralization. Mineralized nodules observed following Alizarin red S staining of undifferentiated cells (**A**,**a**) and of cells at 3 days (**B**,**b**), 7 days (**C**,**c**), 14 days (**D**,**d**), and 21 days (**E**,**e**) after culturing with or without 1 μM arginine (**F**–**J**,**f**–**j**). The line graph shows the staining intensity of Alizarin red S; (**D**) Dose-dependent effect of arginine on mineralization. The bar graph shows the intensity of Alizarin red S staining; (**E**) The mRNA expression of Runx2, DIx5, and osterix transcription factors. Fold-differences were calculated as the relative expression as compared with the expression in control cells. The results are representative of three other experiments, and each bar represents the mean ± S.E.M. ^###^ *p* < 0.001 compared with 0 days; * *p* < 0.05 and *** *p* < 0.001 compared with control.

To determine whether arginine can stimulate osteogenic differentiation, we measured the effect of arginine on the levels of the bone-formation markers type Iα1 collagen, osteocalcin, and alkaline phosphatase (ALP). Our results showed that the treatment of MSCs with 1 μM arginine for 3, 7, 14, and 21 days increased the mRNA expression of type Iα1 collagen, osteocalcin, and ALP in a statistically significant manner, but did not enhance the expression of type IIα1 collagen relative to the control level at each time point (Figure 2A). The expression of type Iα1 collagen peaked between 14 and 21 days during osteogenic differentiation (Figure 2A). In the late stage (after 21 days), the expression of osteocalcin was the highest, 6.5-fold greater than that in control cells (Figure 2A). Furthermore, the expression of ALP was increased by 2.5-, 4.3-, and 4.1-fold relative to control after 7, 14, and 21 days, respectively (Figure 2A). Thus, we further investigated the osteogenic effect of arginine in MSCs. After 14 days of induction, arginine used at concentrations ranging from 0.1–10 μM dose-dependently increased the expression of type Iα1 collagen by 1.4–4.0-fold, of osteocalcin by 1.5–3.7-fold, and of ALP by 2.6–3.2-fold, respectively (Figure 2C). The effect of arginine on osteogenic differentiation, as indicated by extracellular matrix mineralization, was also investigated. After 21 days of treatment, 1 μM arginine increased the matrix calcium deposition by 6.4-fold as compared with the control level (Figure 2B). After 14 days, arginine dose-dependently enhanced mineralization by Iα1 (Figure 2D). To further confirm the osteogenic potential of arginine, we treated MSCs with arginine for 7 days and then measured the mRNA expression of the bone-formation markers Runx2, DIx5, and osterix. Arginine significantly increased the relative mRNA levels of Runx2 by 5.7–6.8-fold, of DIx5 by 1.3–3.7-fold, and of osterix by 3.9–5.1-fold at a dose dependent manner (Figure 2E). In this study, arginine promoted osteogenesis, which was demonstrated by the induction of osteogenic gene-expression markers such as type Iα1 collagen, osteocalcin, and ALP, and eventually stimulated the mineralization of the extracellular matrix. In terms of gene expression levels, treatment with arginine induces the osteoblastic differentiation by increasing the expression of the transcription factors Runx2, DIx5, and osterix. These factors are involved in the decision of a cell to start differentiating into an osteoblast, and the factors play a critical role in stimulating osteoblast-specific gene expression during osteogenesis [8,9,26,27]. The current treatment of osteoporosis relies primarily on antiresorptive agents that inhibit osteoclastic bone resorption and anabolic agents that increase osteoblastic bone formation [2,28], but osteoporosis treatments have exhibited either limited success or adverse effects [29,30]. In this study, arginine enhances osteogenesis without producing side effects, thus we suggest arginine might have substantial potential for reducing osteoporosis by increasing osteogenesis without producing side effects.

### 2.3. Effect of Arginine on Adipogenesis in MSCs

**Figure 3:**
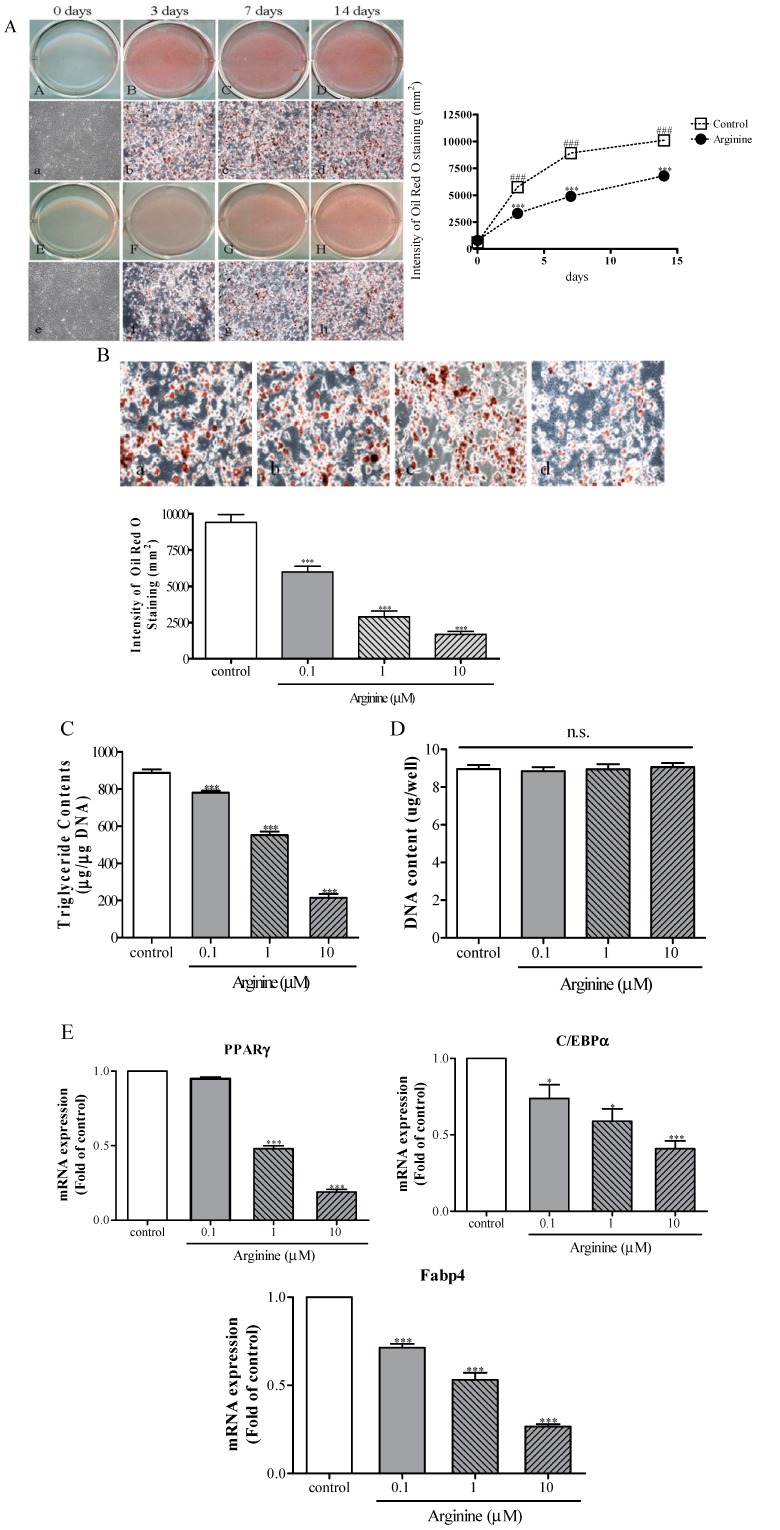
Effect of arginine on adipogenic differentiation in MSCs. (**A**) Temporal effect of arginine on lipid accumulation. MSCs cultured in growth medium were used as the control (**A**,**a**,**E**,**e**), 3 days (**B**,**b**), 7 days (**C**,**c**), and 14 days (**D**,**d**) in differentiation medium or containing 1 μM arginine for 3 days (**F**,**f**), 7 days (**G**,**g**), and 14 days (**H**,**h**). Cells were fixed with 10% formalin and subjected to Oil red O staining. The line graph shows the intensity of Oil red O staining; (**B**) Dose-dependent effect of arginine on lipid accumulation.The bar graph shows the intensity of Oil red O staining; (**C**–**D**) The effect of arginine on TG deposition and DNA content. The cellular TG content was measured at 550 nm using a TG-determination kit (**C**), and DNA content of cells was determined as an internal control (**D**); (**E**) The mRNA expression of PPARγ, CEB/Pα, and Fabp4 transcription factors. The expression levels of all genes were measured using qRT-PCR and normalized relative to the level of β-actin expression. Fold-differences were calculated as the relative expression as compared with the expression in control cells. The results are representative of three other experiments, and each bar represents the mean ± S.E.M. ^###^ *p* < 0.001 compared with 0 days; * *p* < 0.05 and *** *p* < 0.001 compared with control.; n.s., nonsignificant difference compared with control.

To determine the effect of arginine on adipogenic differentiation in MSCs, 1 μM arginine was added to cells cultured in an adipogenic medium, and then adipocyte-specific gene expression was examined using quantitative real-time reverse-transcription PCR (qRT-PCR) during adipogenic induction. During the 14 days of differentiation, the induced adipocytes were stained with Oil red O. The staining-positive cells displayed substantial lipid accumulation at 3 days when compared with the uninduced cells (Figure 3A). The intensity of lipid staining indicated that arginine treatment reduced the rate of adipocytic differentiation in a statistically significant manner by 1.6- and 1.4-fold at 7 and 14 days, respectively, when compared with the control (Figure 3A). To further study the inhibitory effect of arginine on adipogenesis, cells were treated with 0.1, 1, and 10 μM arginine for 14 days, and this treatment inhibited adipocyte formation in MSCs by 1.6-, 3.1-, and 4.5-fold, respectively (Figure 3B); when 1 μM arginine was used, the number of adipocytes was significantly decreased by 69%. Furthermore, the triglyceride (TG) content of arginine-treated cells undergoing adipocytic differentiation was decreased in a dose-dependent manner by 1.1–4.5-fold relative to control (Figure 3C). The DNA content of the cells was not affected by treatment for 14 days with 0.1, 1, and 10 μM arginine, which suggests that arginine inhibited adipogenesis without affecting the cell number (Figure 3D). We determined that in addition to regulating osteogenesis, arginine dose-dependently inhibited adipocyte formation in MSCs. To further confirm the antiadipogenic potential of arginine, we conducted treatment with arginine and then performed qRT-PCR to measure the levels of mRNA expression of the adipocyte-formation markers PPARγ, C/EBPα, and Fabp4. Treatment with arginine lowered the expression of PPARγby 1.2–5.8-fold, of C/EBPα by 1.10–1.8-fold, and of Fabp4 by 1.2–2.5-fold in a dose dependent manner (Figure 3E). In this study, arginine markedly reduced adipocyte formation by inhibiting the expression of the adipogenic transcription factors PPARγ, C/EBPα, and Fabp4 in MSCs. These adipogenic transcription factors act as master regulators of adipogenesis and lipid storage during terminal adipocyte differentiation [10,11]. The complex process of adipogenesis starts with the production of PPARγ, which is controlled and activated by C/EBPα and Fabp4 [16]. *In vivo* studies have demonstrated that in mice in which the PPARγ gene was deleted, the levels of TGs, free fatty acids, and cholesterol and the accumulation of hepatic TG were lower than those in control mice [31]. C/EBPα, which is expressed late in the adipogenesis processes, was reported to promote differentiation by cooperating with PPARγ by means of the cross-regulation mediated by the MEK/ERK signaling pathway [32]. Lipid accumulation within adipocytes is promoted by the gene encoding Fabp4, which is under the transcriptional control of PPARγ [33]. Fabp4 binds to a variety of fatty acids with high affinity and facilitates their storage, tracking, and solubilization [3]. Clinical observations have indicated that in various forms of osteoporosis, the differentiation of MSCs into adipocytes rather than osteoblasts is increased [34]. Our results further demonstrated that arginine in the adipogenic medium caused a decrease in adipocytic differentiation from hMSCs, and the expression of adipogenic marker genes PPARγ, C/EBPα, and Fabp4, and fat accumulation, and triglyceride contents without cytotoxicity, showed a decrease when arginine was present. These results indicate that arginine might contribute to its effects on osteoporosis treatment.

### 2.4. Osteogenic and Adipogenic Differentiation Is Regulated through the NFATc and Wnt Signaling Pathways

**Figure 4:**
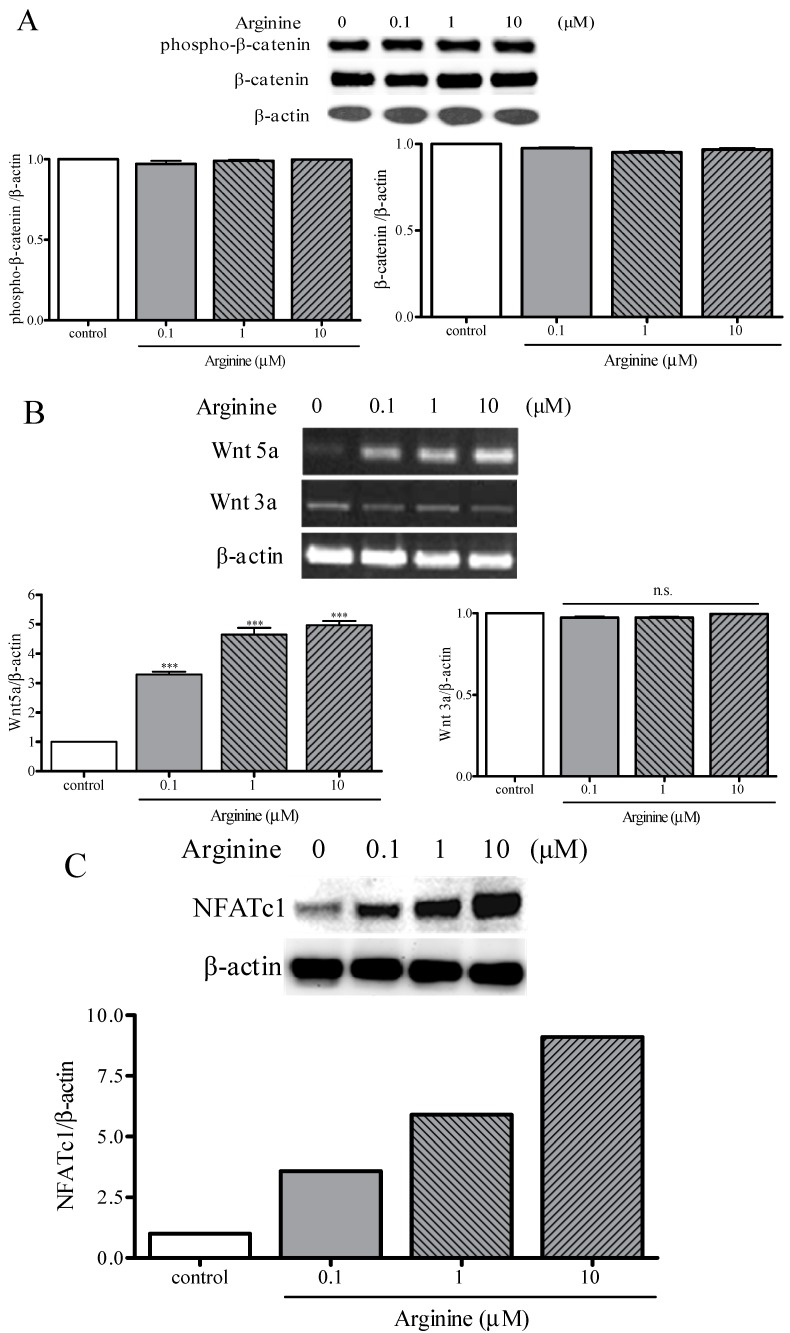
Effect of arginine on the expression of Wnt signaling pathway. (**A**) The representative Western blot analysis for phospho-β-catenin and β-catenin in total protein extracts from MSCs treated indicated dose of arginine for 24 h. The bar graph showed the intensities of phospho-β-catenin and β-catenin expression; (**B**) MSCs were treated with 0, 0.1, 1, and 10 μM arginine for 24 h and then total RNA was isolated and analyzed for Wnt5a, Wnt3a, and β-actin by performing RT-PCR. The bar graphs show the band intensities of Wnt5a and Wnt3a after adjustment for the intensity of β-actin; (**C**) The expression level of NFATc1 was measured using Western blot analysis, and normalized relative to the level of β-actin. Values are mean ± S.E.M. *** *p* <0.001 compared with control; n.s., nonsignificant difference compared with control.

**Figure 5:**
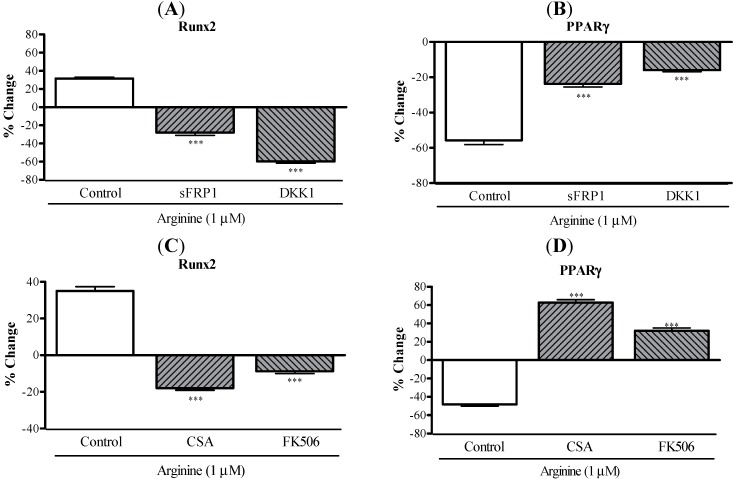
Role of Wnt and NFATc signaling in arginine induced osteoblastic and adipogenic differentiation in MSCs. (**A**,**B**) MSCs were treated with 1 μM arginine in the presence of the Wnt inhibitors sFRP1 (250 ng/mL) and DKK1 (50 ng/mL) and then the expression of Runx2 and PPARγ mRNAs was analyzed using qRT-PCR; (**C**,**D**) MSCs were treated with 1 μM arginine in the presence of calcineurin inhibitors CSA (100 ng/mL) and FK506 (50 ng/mL) and then changes in the expression of Runx2 and PPARγ mRNAs were determined by means of qRT-PCR analysis. The results are representative of two experiments, and each bar represents the mean ± S.E.M. *** *p* < 0.001 compared with control (cells were treated with 1 μM arginine).

To investigate the roles of arginine in Wnt/β-catenin signaling during the osteoblastic and adipocytic differentiation of MSCs, cells cultured in an adipogenic medium were treated with arginine for 48 h and then Western-blotting analysis and RT-PCR were performed. Doses of arginine ranging from 0.1–10 μM did not affect the expression levels of phospho-β-catenin and β-catenin in MSCs (Figure 4A). Subsequently, we analyzed the effect of arginine on the expression of Wnt-family members, which are known to regulate osteogenesis and adipogenesis. Treatment with arginine dose-dependently increased the mRNA level of Wnt5a by 2.1–2.4-fold; by contrast, arginine did not affect the expression of Wnt3a mRNA (Figure 4B). We examined to confirm the role of Wnt5a signaling in mediating the effect of arginine, MSCs treated with 1 μM arginine in the presence of Dickkopf-1 (DKK-1) and secreted Frizzled-related protein-1(sFRP-1), which are recognized inhibitors of Wnt signaling. Addition of DKK1 or sFRP1 abolished arginine induced modulation of Runx2 and PPARγ2 expression in MSCs (Figure 5A,B), which indicates that Wnt signaling partially contributes to the arginine dependent induction of osteoblastogenesis and reduction of adipogenesis in MSCs.

NFATc1 represents a downstream of target of Wnt signaling and is a key regulator of adipogenic and osteogenic differentiation [34,35]. We tested whether arginine acts on MSC adipogenic differentiation by modulating NFATc signaling. Arginine treatment increased the expression of NFATc1 in MSCs in a dose-dependent manner (Figure 4C). When MSCs were treated with arginine in the presence of the calcineurin inhibitor cyclosporine A (CSA) or FK506, arginine induced modulation of Runx2 and PPARγ2 expression in MSCs was abolished (Figure 5C,D). The noncanonical, β-catenin-independent pathway activated by Wnt5a involves several cascades for signal transduction including NFATc [35,36]. Recent studies have indicated that NFATc1 positively controls bone formation through increased osteoblast replication and function and inhibits osteogenic formation [35,36,37,38,39]. Strontium ranelate, a therapeutic agent for osteoporosis, rebalances bone marrow adipogenesis and osteoblastogenesis through NFATc and Wnt signaling in mice [36]. Our study demonstrated that arginine might reverse the impaired bone formation and increased adipogenesis in MSCs through the regulation of NFATc1 by activation of Wnt5a. Arginine has been shown to trigger substantial nitric oxide (NO) synthesis by activation of inducible NO synthetase (iNOS) [21]. As has been recently reported, Wnt5a signaling is related to NO production, which in turn increases NMDA receptor trafficking to the cell surface [40,41]. The regulation of Wnt5a expression might be mediated by NO, but the mechanism by which NO could mediate β-catenin-dependent or β-catenin-independent stabilization remains unknown. This warrants further investigation.

Taken together, our results suggest that arginine regulates Wnt/β-catenin independent signaling and also the mechanisms involving NFATc that control MSC fate and differentiation, and contributes to the increase in osteoblastogenesis and decrease in adipogenesis.

## 3. Materials and Methods

### 3.1. Chemicals and Reagents

l-Arginine, ALP activity assay kit, Alizarin red S staining kit, Oil red O staining kit, ascorbic acid, glycerophosphate, dexamethasone (DEX), 3-isobutyl-1-methylxanthine (IBMX), dimethyl sulfoxide (DMSO), and insulin (INS) were purchased from Sigma-Aldrich (St Louis, MO, USA). Fetal bovine serum (FBS), fetal calf serum (FCS), antibiotics, Dulbecco’s modified Eagle’s medium (DMEM), Trizol R reagent, and SDS-polyacrylamide gels were purchased from Gibco-BRL (now part of Invitrogen Corporation; Carlsbad, CA, USA). The RT-PCR system kit was purchased from TaKaRa Biotechnology (Seoul, Korea). The real-time SYBR Green RT-PCR system kit was purchased from Bio-Rad (Roche Diagnostics, Mannheim, Germany). Hybond-C nitrocellulose membrane was purchased from Amersham Biosciences (Piscataway, NJ, USA).

### 3.2. Cell Culture and Treatments

Human bone-marrow MSCs were obtained from Lonza (ATCC CL-173™; Manassas, VA, USA). Cells were seeded into 25-cm^2^ flasks and incubated at 37 °C/5% CO_2_. After 48 h, non-adherent cells were removed from the flasks by changing the medium. Thereafter, the medium was changed once every 3 days. Typically, cultures reached 90% confluence by 14 days, at which point the cells were trypsinized using 0.25% trypsin-0.53 mM EDTA, counted, and then plated again. Cells from passages number of 4~6 were used in all experiments. To determine whether changes in gene expression depended on canonical or noncanonical Wnt signaling, MSCs were treated with arginine in the presence of DKK1 (50 ng/mL) or sFRP1 (250 ng/mL) (Sigma) and then gene expression was measured as described below (in RT-PCR and qRT-PCR analysis). To investigate the role of NFATc in mediating the effect of arginine, MSCs were treated with arginine in the presence of CSA (100 ng/mL) or FK506 (10 ng/mL), and then gene expression was measured.

### 3.3. Cell Proliferation Assay

MSCs were seeded at a density of 2 × 10^4^ cells/well in 96-well plates and allowed to attach for 12 h in DMEM containing 10% FBS. To assess the effect of arginine on cell proliferation, MSCs were treated with culture medium containing various concentrations of arginine (0, 0.01, 0.1, 1, 10, 100 μM) for 48 h or with medium containing 0, 0.1, 1, and 10 μM arginine for 3, 5, 7, and 10 days. At each time point, the arginine containing medium was removed and the cells were incubated with fresh serum-free medium containing the Cell Counting Kit-8 (CCK-8) reagent WST-8 (2-(2-methoxy-4-nitrophenyl)-3-(4-nitro-phenyl)-5-(2,4-disulfophenyl)-2H-tetrazolium) (DOJINDO Lab., Tokyo, Japan); cells were incubated for 2 h at 37 °C in a CO_2_ incubator and then the amount of formazan dye generated, which is proportional to the number of living cells, was determined by measuring the absorbance at 570 nm using a multi-well plate reader (Molecular Devices Co., Sunnyvale, CA, USA). The percentage of proliferating cells was calculated by defining the cell viability measured in the absence of treatment as 100%.

### 3.4. Assessment of Osteogenic Differentiation

To induce osteogenic differentiation, MSCs were plated at a density of 2 × 10^4^ cells/cm^2^ in 6-well plates. After 2 days, the medium was replaced with an osteogenic medium (low-glucose DMEM containing 5% FCS, 10 nM DEX, 50 μM l-ascorbic acid-2-phosphate, and 10 mM glycerophosphate), and then fresh differentiation medium was added every other day and the cells were maintained at 37 °C in a humidified 5% CO_2_ atmosphere throughout the experiments until the cells were harvested. The differentiating cells were treated for 3, 7, 14, and 21 days with the osteogenic differentiation medium containing arginine at a final concentration of 1 μM, or the cells were treated with 0.1, 1, and 10 μM arginine. To determine the effects of arginine on osteogenesis in MSCs, the expression of osteogenesis-related biochemical markers such as collagen type 1α, osteocalcin, and ALP and transcription factors such as Runx2, DIx5, and osterix were measured using qRT-PCR and matrix calcium deposition was evaluated by performing Alizarin red S staining.

### 3.5. Alizarin Red S Staining

After treatment with arginine for 21 days, mineralization was measured using Alizarin red S (Sigma) staining and phase-contrast microscopy. Cells were incubated with 2% Alizarin red at pH 4.2 for 10 min and then washed with distilled water; the sub-cultured cells were observed using phase-contrast microscopy to examine cell morphology and verify the presence of mineralized nodules. After staining, the cells were washed and photographed, and the dye retained by the cells was eluted by incubation with isopropanol and then quantified by measuring the absorbance at 510 nm.

### 3.6. Assessment of Adipogenic Differentiation

MSCs were plated at a density of 3 × 10^4^ cells/cm^2^ in 6-well plates and grown to post-confluence for 3 days. The culture medium was subsequently replaced with the adipogenesis-induction medium (high-glucose DMEM containing 5% FBS, 1 μM DEX, 50 μg/mL INS, and 0.5 mM IBMX) in order to commit the cells to the adipogenic lineage. This medium was changed every other day and maintained up to 14 days to ensure complete development of the adipocyte phenotype. To assess the effect of arginine, cells were treated for 3, 7, and 14 days with the adipocytic differentiation medium containing arginine at a final concentration of 1 μM, or cells were treated with 0.1, 1, and 10 μM arginine for 14 days, and then the cells were used for qRT-PCR and RT-PCR assays, Oil red O staining, and TG-content measurement. The expression of Wnt3a, Wnt5a, and β-catenin was also evaluated during the adipocytic differentiation of MSCs.

### 3.7. Oil Red O Staining

Intracellular lipid accumulation was measured using Oil red O staining; the staining of lipid droplets by this dye was used as an indicator of the degree of adipogenesis. Cells were carefully washed twice with phosphate-buffered saline (PBS) and then fixed and dried with 10% formalin for 20 min. Next, the 10% formalin was removed and 60% isopropanol was added to each well for 3 min and then the cells were washed thoroughly with PBS to remove unbound dye. Cells were incubated with the Oil red O solution for 20 min, and the staining of lipid droplets in differentiated MSCs was examined after rinsing the cells 3 times with distilled water. Cells were photographed, and then the dye retained by the cells was eluted by incubating the cells with isopropanol, and quantified by measuring the absorbance at 510 nm.

### 3.8. TG Assay and DNA-Content Measurement

The cellular content of TG was measured using a TG-determination kit (Wako Corp., Osaka, Japan). Briefly, the cells were rinsed 3 times with PBS and then scraped off the plate by using a rubber policeman. The cells were extracted using 1 mL of lysis buffer (50 mM Tris, 0.15 M NaCl, 10 mM EDTA, 0.1% Tween-20, pH 7.5 set with HCl) and then sonicated for 30 s at 4 °C. Next, 20 μL of the cellular lysate was mixed with 3 mL of the enzyme solution supplied with the kit, and the mixture was incubated for 10 min at 37 °C. The absorbance at 550 nm was measured within 60 min. As an internal control, the DNA concentration in MSCs was determined using spectrophotometry and then the μg/μg value was calculated.

### 3.9. Quantitative Real-Time Polymerase Chain Reaction (qRT-PCR) and Reverse Transcriptase-Polymerase Chain Reaction (RT-PCR).

Total cellular RNA was extracted from osteogenic cells or adipocytes by using the Trizol reagent and then centrifuged at 12,000 rpm for 10 min at 4 °C. Next, 1 μg of total RNA was reverse transcribed into cDNA for 60 min at 42 °C and then for 15 min at 72 °C by using an RT-PCR mixture (TaKaRa Biotechnology) that contained the RT buffer, oligo(dT) 12-mer, 10 mM dNTPs, 0.1 M dithiothreitol, reverse transcriptase, and RNase inhibitor. The qRT-PCR assay was performed in a 25-μL reaction mixture containing the SYBR Green PCR Master Mix (Roche Diagnostics). The template source was either 5 ng of cDNA or a purified DNA standard. Various primers were used for amplifying type IIα1 collagen, type Iα1 collagen, osteocalcin, ALP, Runx2, DIx5, osterix, PPARγ, C/EBPα, and Fabp4. To standardize mRNA levels, we amplified β-actin as an internal control. The relative expression of target genes in the examined samples was obtained using the difference in the comparative threshold (*C*_t_) method. The cycle of threshold (*C*_t_) for each sample was averaged and normalized relative to that of GAPDH. The results were then analyzed using the comparative ΔΔ*C*_t_ method (2^(−ΔΔ*C*t)^) for the purpose of quantifying relative gene-expression levels. To perform RT-PCR, the diluted first-strand cDNA samples were amplified using TaqDNA polymerase (TaKaRa Biotechnology). The mRNA expression levels of Wnt5a, Wnt3a, and β-actin were evaluated by performing ethidium-bromide staining of PCR products. The signal intensity was quantified using Gel Doc EQ (BIO-RAD Laboratories, Milan, Italy), and the relative expression of the mRNAs was normalized by dividing the signal of the target genes by that of the respective β-actin sample. The sequences of the primers used for qRT-PCR and RT-PCR assays are listed in Table 1 and Table 2.

**Table 1:** Primer design for quantitative real-time reverse transcriptase-polymerase chain reaction (qRT-PCR) analysis.

mRNA	Primers	Annealing Tm (Cycle)	
Type II α1 collagen	Fw: 5'-AACACTGCAACGTCCAGAT-3'	58 °C (32)	
	Rv: 5'-CTGCAGCACGGTATAGGTGA-3'		
Type I α1 collagen	Fw: 5'-TGACCTCAAGATGTGCCACT-3'	58 °C (32)	
	Rv: 5'-GGGAGTTTCCATGAAGCCAC-3'		
Osteocalcin	Fw: 5'-CATGAGAGCCCTCACA-3'	55 °C (32)	
	Rv: 5'-AGAGCGACACCCTAGAC-3'		
Alkaline Phosphatase	Fw: 5'-TCAGAAGCTCAACACCAACG-3'	58 °C (32)	
	Rv: 5'-GTCAGGGACCTGGGCATT-3'		
Runx2	Fw: 5'-ACAACCACAGAACCACAAG-3'	58 °C (30)	
	Rv: 5'-TCTCGGTGGCTGGTAGTGA-3'		
DIx5	Fw: 5'-GACAGGATCCCTATGACAGGAGTG-3'	58 °C (30)	
	Rv: 5'-GGACTCGAGATCTAATAAAGCGTC-3'		
Osterix	Fw: 5'-TGAGGAAGAAGCCCATTCAC-3'	58 °C (30)	
	Rv: 5'-ACTTCTTCTCCCGGGTGTG-3'		
Proliferators-activated receptor γ	Fw: 5'-AGAACACCTCTGAAAGTAAG-3'	58 °C (30)	
	Rv: 5'-ACTGTGATGTACTGCTGAAC-3'		
CCAAT/enhancer binding protein α	Fw: 5'-GAGTGACAAGCCTGTAGCC-3'	58 °C (30)	
	Rv: 5'-GGTTGACTTTCTCCTGGTAT-3'		
Fatty acid binding protein 4	Fw: 5'-TCAGTTCGTCCTCACTCCAG-3'	58 °C (30)	
	Rv: 5'-TTGGTCCACCTGTCATCTTC-3'		
β-actin	Fw: 5'-GCTCTCCAGAACATCACTCCTGCC-3'	58 °C (30)	
	Rv: 5'-CGTTGTCATACCAGGAAATGAGCTT-3'		

Fw, forward; Rv, reverse; Tm, temperature.

**Table 2:** Primer design for RT-PCR analysis.

mRNA	Primers	Annealing Tm (Cycle)
Wnt5a	Fw: 5'-ACGCTA AGGGTTCCTATGAG-3'	58 °C (32)
	Rv: 5'-CATAGCAGCACCAGTGAAAC-3'	
Wnt3a	Fw: 5'-GTTCTGCAGCGAAGTGGTG-3'	58 °C (32)
	Rv: 5'-CTGCAGCACGGTATAGGTGA-3'	
β-actin	Fw: 5'-GCTCTCCAGAACATCACTCCTGCC-3'	58 °C (30)
	Rv: 5'-CGTTGTCATACCAGGAAATGAGCTT-3'	

Fw, forward; Rv, reverse; Tm, temperature.

### 3.10. Western Blotting

Following incubation with various reagents, cells were lysed using the lysis buffer. Total proteins (20 μg/lane) were separated on 4%–12% SDS-polyacrylamide gels by preforming electrophoresis under reducing conditions, and then transferred to Hybond-C nitrocellulose membranes (Amersham Biosciences) at 300 mA for 90 min. After blocking with 5% non-fat skim milk for 2 h, the membranes were incubated with rabbit primary antibodies against phospho-β-catenin, anti-β-catenin and NFATc1 (1:1000 dilution; Santa Cruz Biotechnology, Santa Cruz, CA, USA) for 12 h at 4 °C. The membranes were washed 4 times in 1× TTBS (Tween^®^/Tris-buffered saline), 5 min per wash, on a shaker. Next, the blots were incubated with goat anti-rabbit IgG-HRP (1:2000; Santa Cruz Biotechnology) for 2 h at room temperature, and then washed 4 times in TTBS, 5 min each time. Immunoreactive proteins on the blots were visualized using ECL™ Western Blotting detection reagents and the signals were detected using Image Station 4000R (Kodak, New Haven, CT, USA). Staining with an anti-β-actin antibody was used to verify that equal amounts of proteins were loaded in all lanes.

### 3.11. Statistical Analysis

Values are expressed as means ± standard error of the mean (S.E.M). In the case of two groups, Student’s *t* tests were performed, or differences among groups were analyzed by means of one-way ANOVA performed to evaluate statistical significance by using the Prism 5.0 program. Differences were considered statistically significant at a level of *p* < 0.05.

## 4. Conclusions

**Figure 6:**
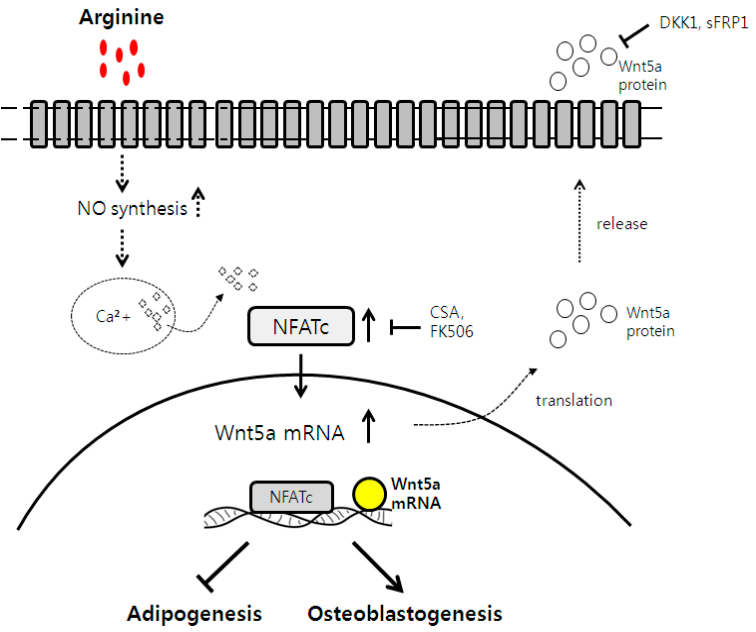
Proposed mechanism of pro-bone and anti-fat effect of arginine, mediated NO synthesis and induced NFATc accumulation, and subsequent increased Wnt5a mRNA expression in human mesenchymal stem cells. 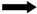 Indicates activation or induction; 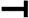 indicates inhibition or blockade.

In conclusion, this study has presented the first experimental evidence of the pro-bone and anti-fat effects of arginine, which counteracts the age-related switch in MSC osteoblast-to-adipocyte differentiation through the Wnt and NFATc1 signaling pathways. A schematic diagram of these observed effects of arginine is shown in Figure 6. These findings indicate that arginine supplement offers great promise for the prevention and treatment of osteoporosis.
